# Diffusion Barrier Performance of Ni-W Layer at Sn/Cu Interfacial Reaction

**DOI:** 10.3390/ma17153682

**Published:** 2024-07-25

**Authors:** Jinye Yao, Chenyu Li, Min Shang, Xiangxu Chen, Yunpeng Wang, Haoran Ma, Haitao Ma, Xiaoying Liu

**Affiliations:** 1 School of Materials Science and Engineering, Dalian University of Technology, Dalian 116000, China; yaojinye@mail.dlut.edu.cn (J.Y.); lcxlcy66@mail.dlut.edu.cn (C.L.); shangxiaomin2019@mail.dlut.edu.cn (M.S.); xiangxuchen@mail.dlut.edu.cn (X.C.); yunpengw@dlut.edu.cn (Y.W.); 2 School of Microelectronics, Dalian University of Technology, Dalian 116024, China; mhr@dlut.edu.cn

**Keywords:** Ni-W layer, electrodeposition, intermetallic compounds, interfacial reaction, diffusion

## Abstract

As the integration of chips in 3D integrated circuits (ICs) increases and the size of micro-bumps reduces, issues with the reliability of service due to electromigration and thermomigration are becoming more prevalent. In the practical application of solder joints, an increase in the grain size of intermetallic compounds (IMCs) has been observed during the reflow process. This phenomenon results in an increased thickness of the IMC layer, accompanied by a proportional increase in the volume of the IMC layer within the joint. The brittle nature of IMC renders it susceptible to excessive growth in small-sized joints, which has the potential to negatively impact the reliability of the welded joint. It is therefore of the utmost importance to regulate the formation and growth of IMCs. The following paper presents the electrodeposition of a Ni-W layer on a Cu substrate, forming a barrier layer. Subsequently, the barrier properties between the Sn/Cu reactive couples were subjected to a comprehensive and systematic investigation. The study indicates that the Ni-W layer has the capacity to impede the diffusion of Sn atoms into Cu. Furthermore, the Ni-W layer is a viable diffusion barrier at the Sn/Cu interface. The “bright layer” Ni_2_WSn_4_ can be observed in all Ni-W coatings during the soldering reflow process, and its growth was almost linear. The structure of the Ni-W layer is such that it reduces the barrier properties that would otherwise be inherent to it. This is due to the “bright layer” Ni_2_WSn_4_ that covers the original Ni-W barrier layer. At a temperature of 300 °C for a duration of 600 s, the Ni-W barrier layer loses its blocking function. Once the “bright layer” Ni_2_WSn_4_ has completely covered the original Ni-W barrier layer, the diffusion activation energy for Sn diffusion into the Cu substrate side will be significantly reduced, particularly in areas where the distortion energy is concentrated due to electroplating tension. Both the “bright layer” Ni_2_WSn_4_ and Sn will grow rapidly, with the formation of Cu-Sn intermetallic compounds (IMCs). At temperatures of 250 °C, the growth of Ni_3_Sn_4_-based IMCs is controlled by grain boundaries. Conversely, the growth of the Ni_2_WSn_4_ layer (consumption of Ni-W layer) is influenced by a combination of grain boundary diffusion and bulk diffusion. At temperatures of 275 °C and 300 °C, the growth of Ni_3_Sn_4_-based IMCs and the Ni_2_WSn_4_ layer (consumption of Ni-W layer) are both controlled by grain boundaries. The findings of this study can inform the theoretical design of solder joints with barrier layers as well as the selection of Ni-W diffusion barrier layers for use in different soldering processes. This can, in turn, enhance the reliability of microelectronic devices, offering significant theoretical and practical value.

## 1. Introduction

The demand for the miniaturization, portability, and multifunctionality of electronic products has driven a significant increase in the packaging density of electronic devices [1,2,3]. This has led to the development of electronic packaging technology from 2D to 3D, with a concomitant reduction in the size of solder joints [4,5,6]. As the dimensions of the solder joints diminish, the electric, thermal, and mechanical forces exerted on the solder joints increase, rather than decrease, and the reliability of the solder joints faces significant challenges [7,8]. Furthermore, the solder joint interface element interaction, diffusion enhancement, and electromigration are intensified by multiple effects including those of the solder joint soldering reflow process of the intermetallic compound (IMC) grain size [9]. This results in an increase in the thickness of the IMC layer and a corresponding increase in the proportion of the volume of the solder joints accounted for by the IMC layer. The brittleness of the IMC renders it susceptible to overgrowth in small-sized solder joints, which in turn compromises the reliability of the solder joints [10,11]. It is therefore of great importance to regulate the formation and growth of IMCs.

The incorporation of a diffusion barrier layer within the solder joint structure represents an effective means of addressing the potential reliability issues that may arise as a consequence of IMC overgrowth [12]. The diffusion barrier should possess the following characteristics: the diffusion barrier should be capable of maintaining a robust bond with the microbump copper and solder, should not dissolve during the reflow process, and should exhibit good solderability [13,14,15]. The classification of diffusion barriers is based on the type of material from which they are constructed. These barriers can be classified into five categories: simple substances, binary compounds, ternary compounds, composites, and multilayer membrane structures [16,17]. Ni is an optimal material for use as a diffusion barrier, exhibiting both high bonding strength and long-lasting diffusion effects [18,19]. Consequently, the employment of Ni as a diffusion barrier has been demonstrated to effectively inhibit the excessive growth of IMCs. The incorporation of Ni can impede the interaction between Cu elements in Cu substrates and Sn-based soldering materials [20,21]. In the solder joints of the actual service temperature conditions, the atomic diffusion of Ni to the grain boundary diffusion is dominated by Ni in the Cu diffusion rate of 2.9 × 10^−6^ cm^2^/s and Cu in the Ni diffusion rate of 1.7 × 10^−6^ cm^2^/s. Nevertheless, despite the two being in the same order of magnitude, the diffusion rate of the two metal atoms will significantly increase with an increase in the density of the circuit [22]. This results in the Ni being eroded by the Cu, which causes electromigration holes. It is therefore essential to incorporate refractory metal elements within the Ni blocking layer with the objective of reducing the diffusion rate of Ni [23].

The incorporation of metal W into the coating results in enhanced thermal stability and mechanical properties when compared to pure nickel coatings [24]. Consequently, Ni-W alloy coatings exhibit excellent corrosion and wear resistance, hardness, and other properties [25,26]. Further systematic studies are required to investigate the interfacial reaction behavior between Ni-W coatings and lead-free solder and to assess the feasibility of Ni-W coatings as brazing diffusion barrier layers. In this investigation, an electrodeposition process was employed to deposit a Ni-W coating on a Cu substrate. The objective of this study was to examine the wetting behavior between molten Sn and Ni-W/Cu substrates and present a discussion of the evolution of the microstructure of the Sn/Ni-W/Cu interface and the depletion behavior of the Ni-W barrier layer. The findings of this study can inform the theoretical design of solder joints with a Ni-W diffusion barrier layer.

## 2. Materials and Methods

Prior to electrodeposition, the Cu substrates, with a purity of 99.95%, had a dimension of 200 mm × 200 mm × 1 mm and underwent sanding and polishing steps. Subsequently, a series of cleaning and activation steps were undertaken. The process comprised an acid wash with a 5% HCl solution (to eliminate surface oxides), a deionized water wash, sensitization with SnCl_2_ (10 g/L), and activation with 15% H_2_SO_4_ (to create specific sensitized, activated centers on the surface, which facilitate the subsequent nucleation of the plating). The electrodeposition of Ni-W coatings is characterized by a significant disparity in atomic radii between Ni and W, which gives rise to a rapid deposition rate. However, this also results in the generation of substantial internal stresses, which can lead to the formation of surface cracks in the coating [27]. Consequently, ultrasonic assistance was employed during the electrodeposition of Ni-W in order to eliminate internal stresses and suppress surface cracks.

The composition of the electrodeposition solution is presented in Table 1. The electrodeposition time and temperature were 60 min and 65 °C, respectively. The pH was set to 7.5, the current density was 1.5 A/dm^2^, and the ultrasonic frequency was 80 kHz. The solder balls, with a diameter of 1000 μm, were attached to Ni-W/Cu substrates at temperatures of 250, 275, and 300 °C. The solder balls were composed of pure Sn. The interfacial IMCs were chemically etched using a solution comprising 5 vol.% HNO_3_, 2 vol.% HCl, and 93 vol.% CH_3_OH.

**Figure 1:**
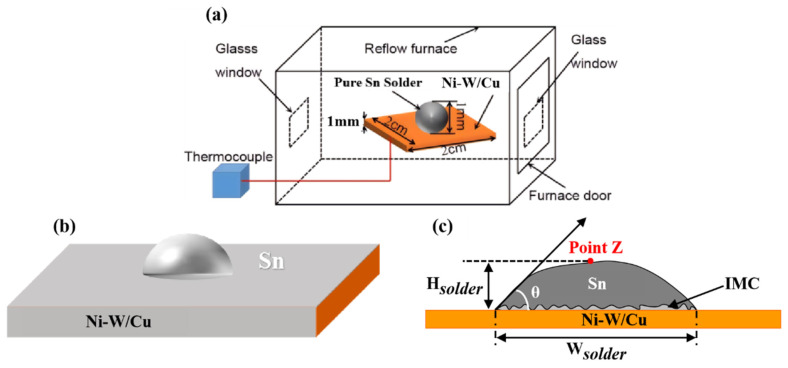
Schematic of the soldering reflow experiment: (**a**) soldering process; (**b**) interface reaction of the pure Sn/Ni-W/Cu substrates; (**c**) wetting angle measurement.

Figure 1 depicts the process flow diagram of soldering. The morphological and compositional characteristics of the Sn/Ni-W/Cu substrates and IMCs were verified through the utilization of a secondary electron microscope (SEM, SUPARR 55, Zeiss Optical Instruments Co., Ltd., Oberkochen, Germany) and electron probe X-ray microanalyzer (EPMA, JXA-8530F PLUS, Japan Electron Optics Laboratory Co., Ltd., Tokyo, Japan). The wetting rate was quantified utilizing an SDC 350 contact angle measuring instrument (Shengding Precision Instrument Co., Ltd., Dongguan, China).

## 3. Results and Discussion

### 3.1. Characterization of Ni-W Diffusion Barrier Layer

**Figure 2:**
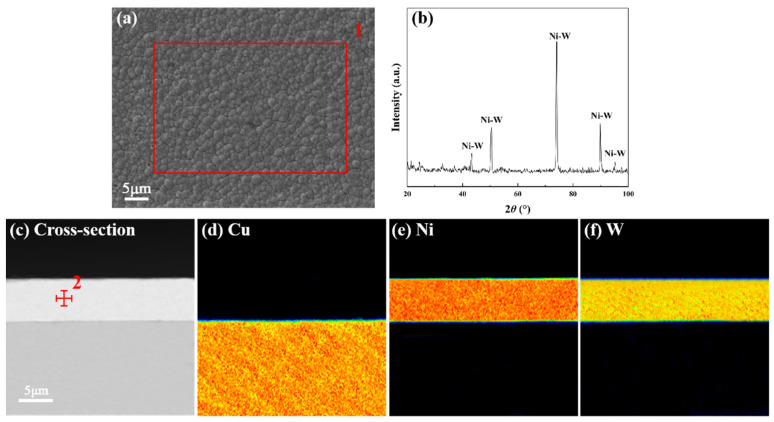
(**a**) Top-view SEM images of the Ni-W coating; (**b**) XRD pattern of the Ni-W coating; (**c**) Cross-sectional SEM images of the Ni-W/Cu substrate. (**d**–**f**) The corresponding EDS maps of the cross-sectional SEM images of the Ni-W/Cu substrate.

Figure 2a illustrates the typical cellular morphology of the Ni-W particles. The substitution of W for Ni resulted in the formation of localized distortions around the lattice of the W atoms. Figure 2b illustrates the X-ray diffraction pattern of the Ni-W coating, which exhibited no diffraction peaks associated with the Cu substrate. This observation indicates that the coating had a greater thickness and had completely encapsulated the Cu substrate. As depicted in Figure 2c, the thickness of the Ni-W diffusion barrier layer was found to be 6.0 ± 0.2 μm. As illustrated in Figure 2d–f, the Ni-W coating exhibited uniform and dense characteristics throughout the cross-section. The Cu substrate and the Ni-W barrier layer were continuous and exhibited no undulations. Furthermore, no discernible instances of delamination, looseness, holes, or other defects were observed. This indicates that the Cu substrate and Ni-W diffusion barrier layer exhibited excellent bonding characteristics. Table 2 indicates that the W content of the Ni-W layer was approximately 25 wt.%.

### 3.2. Microstructure Evolution of IMCs

**Figure 3:**
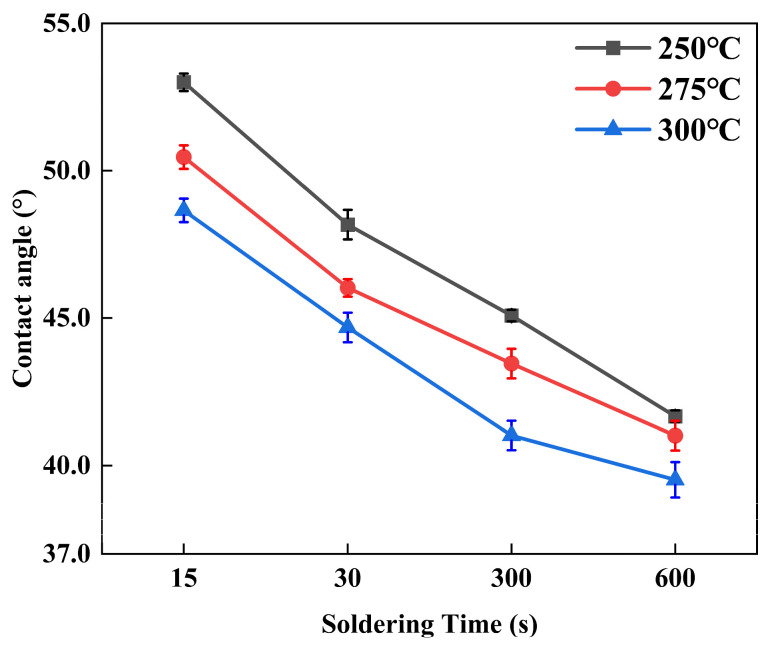
Contact angle of the molten pure Sn on Ni-W/Cu substrates under different soldering conditions.

Figure 3 illustrates that the variation in the wetting angle of molten pure Sn on a Ni-W/Cu substrate varies under different soldering conditions. A reduction in the contact angle is indicative of an enhanced wettability between the molten solder balls and the substrate [28]. As shown in Figure 3, the contact angle exhibited a decreasing trend with an increase in welding time at a constant welding temperature. Furthermore, an increase in the temperature of the weld at a constant weld time also resulted in a reduction in the contact angle. This phenomenon can be attributed to the fact that the surface tension of the molten Sn balls increases in proportion to the ambient temperature, resulting from the thermal vibration of the metal atoms within. The wetting angle of the Sn/Ni-W/Cu solder joints in this soldering process was found to be within the range of 39°–53°. It is postulated that within this range, the wettability of the Ni-W/Cu substrate is satisfactory [29].

**Figure 4:**
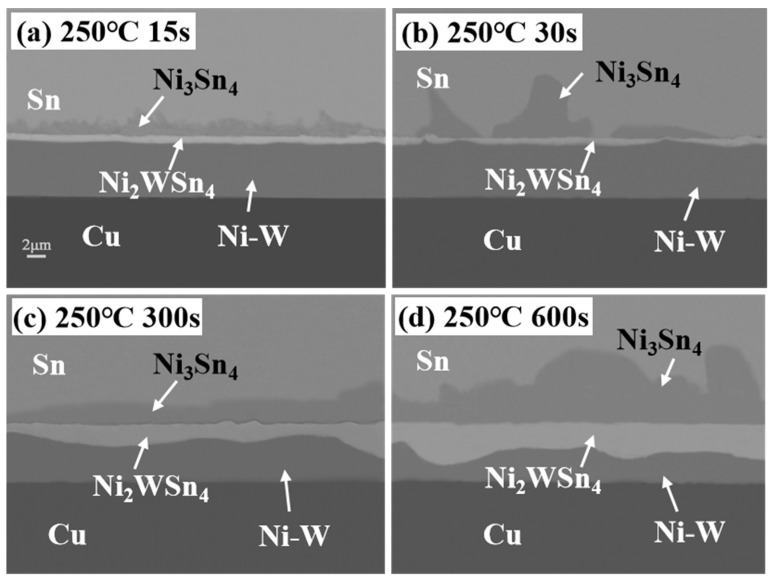
Cross-sectional SEM images of Sn/Ni-W/Cu substrates soldered for different periods of time at 250 °C: (**a**) 15 s; (**b**) 30 s; (**c**) 300 s; (**d**) 600 s.

**Figure 5:**
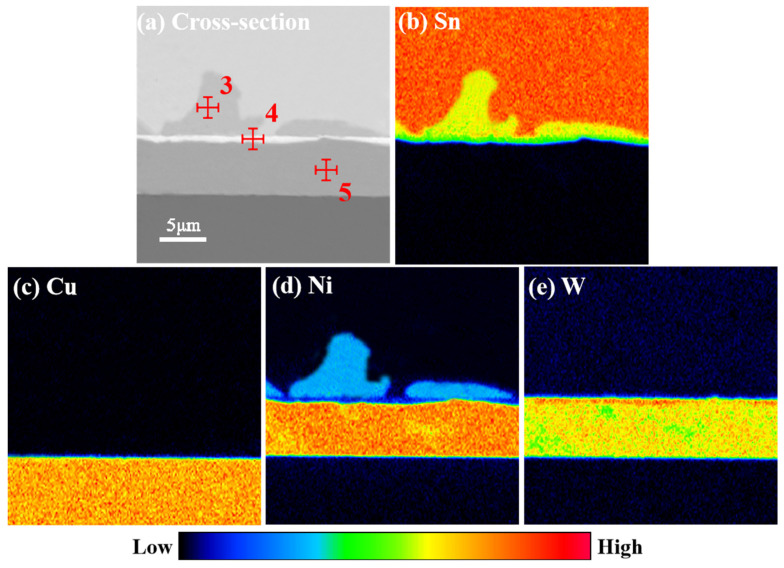
EDS maps of Sn/Ni-W/Cu substrates soldered for 30 s at 250 °C.

Figure 4 illustrates that following refluxing at 250 °C for varying periods, the pure Sn soldering material underwent a reaction with the Ni-W diffusion barrier layer, resulting in the formation of the Ni_3_Sn_4_ IMC. The thickness of the Ni_3_Sn_4_ IMC increased gradually with the extension of time. Additionally, a continuous “bright layer” could be observed on the lower side of the Ni_3_Sn_4_ IMC. The bright part of the original Ni-W diffusion barrier layer, which was evidently distinct from the surrounding phase, was designated as the “bright layer”. This phenomenon can be attributed to the fact that at the outset of the interfacial reaction, Ni-W reacts with liquid Sn, leading to the formation of a continuous layer of the Ni_3_Sn_4_ IMC phase. Subsequently, the Ni atoms in the Ni-W solid solution in the Ni-W diffusion barrier layer continue to react by diffusion into pure Sn, resulting in a notable increase in the W content at the top of the alloy layer in direct contact with the pure Sn solder. Figure 5 illustrates the EDS maps of Sn/Ni-W/Cu substrates that had undergone soldered for 30 s at 250 °C. The shift from dark blue to red in the graph reflects a gradual increase in concentration. In the diagram illustrating the distribution of Sn, the red color represents pure Sn, the yellow area represents Ni_3_Sn_4_, and the green area represents “bright layer” Ni_2_WSn_4_. In the diagram showing the distribution of Cu, the orange color represents the Cu substrate. In the diagram showing the distribution of Ni, the light blue color is Ni_3_Sn_4_, the dark blue color is the “bright layer” Ni_2_WSn_4_, and the orange color represents the Ni-W layer. In the W distribution diagram, the red area is the “bright layer” Ni_2_WSn_4_, and the yellow area represents the Ni-W layer. As illustrated in Figure 5 and Table 3, a continuous layer formed at the Ni-W diffusion barrier layer/pure Sn solder interface, comprising 29% at.%Ni, 14% at.%W, and 57% at.%Sn. It is reasonable to posit that the “bright layer” is Ni_2_WSn_4_. The Ni_2_WSn_4_ layer further impeded the further diffusion of Ni in the Ni-W diffusion barrier layer. As the soldering time increased, the thickness of the Ni_3_Sn_4_ IMC, which formed between the Sn and Ni-W diffusion barrier layer, also increased. Concurrently, the thickness of the “bright layer” Ni_2_WSn_4_ also increased. This process occurred gradually, with the Ni-W diffusion barrier layer becoming progressively covered. Concurrently, it was observed that the thickness of the original Ni-W diffusion barrier layer underwent a notable reduction. It can be reasonably assumed that Ni_2_WSn_4_ is generated at the same rate as the original Ni-W diffusion barrier layer is consumed.

**Figure 6:**
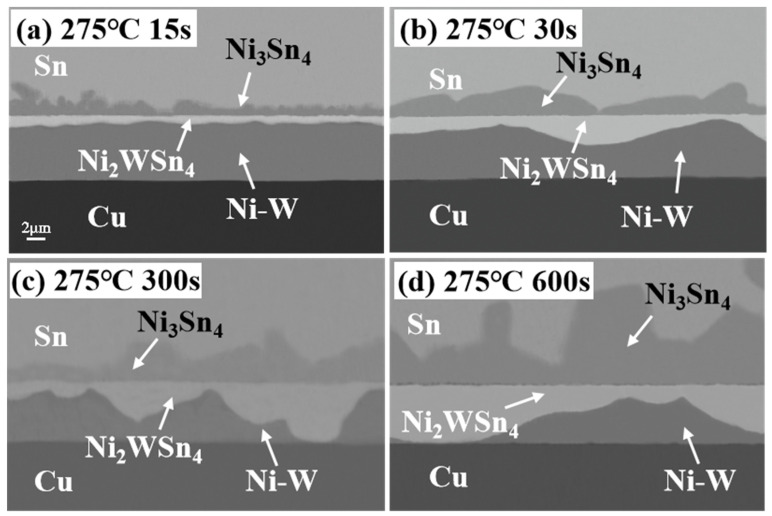
Cross-sectional SEM images of Sn/Ni-W/Cu substrates soldered for different periods of time at 275 °C: (**a**) 15 s; (**b**) 30 s; (**c**) 300 s; (**d**) 600 s.

**Figure 7:**
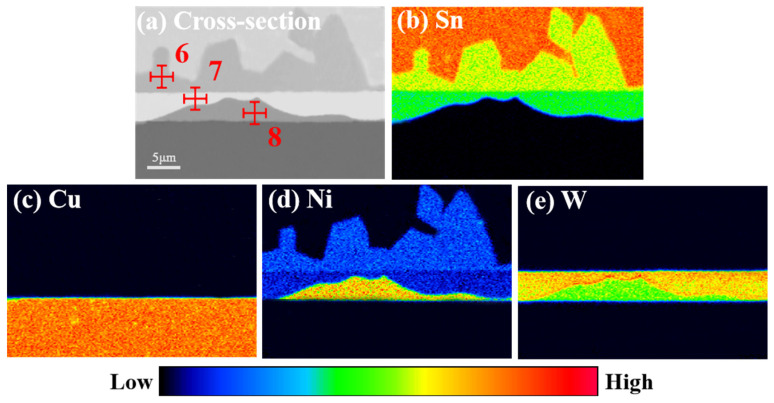
EDS maps of Sn/Ni-W/Cu substrates soldered for 600 s at 275 °C.

The microstructure of the Sn/Ni-W/Cu substrates soldered for different times at 275 °C is presented in Figure 6. The matrix Cu did not react with the pure Sn solder at this temperature, as evidenced by the lack of any observed reaction. Upon soldering the Sn/Ni-W/Cu substrates at 275 °C, the Ni-W diffusion barrier layer began to decompose, with Sn diffusing into the diffusion barrier layer. The IMC layer, comprising Sn and Ni atoms, underwent a diffusion process to form the Ni_3_Sn_4_ IMC. The thickness of the Ni_3_Sn_4_ IMC exhibited a gradual increase over time. Furthermore, a continuous layer of “bright layer” Ni_2_WSn_4_ could be observed on the lower side of the Ni_3_Sn_4_ IMC. The thickness of the Ni_3_Sn_4_ IMC and “bright layer” Ni_2_WSn_4_ increased at 275 °C in comparison to 250 °C. Figure 7 depicts the EDS maps of the Sn/Ni-W/Cu substrates soldered for 600 s at 275 °C. The transition from dark blue to red in the graph represents a gradual increase in concentration. The distribution diagram of Sn exhibited a red color representing pure Sn, a yellow area representing Ni_3_Sn_4_, and a green area representing the “bright layer” Ni_2_WSn_4_. Similarly, the distribution diagram of Cu displayed an orange color representing the Cu substrate. In the Ni distribution diagram, the light blue color is Ni_3_Sn_4_, the dark blue color is the “bright layer” Ni_2_WSn_4_, and the orange color represents the Ni-W layer. In the W distribution diagram, the orange area is the “bright layer” Ni_2_WSn_4_, and the green area represents the Ni-W layer. As illustrated in Figure 7 and Table 4, at 275 °C for 300 s and 600 s, a significant portion of Ni_2_WSn_4_ had completely replaced the original Ni-W diffusion barrier layer.

**Figure 8:**
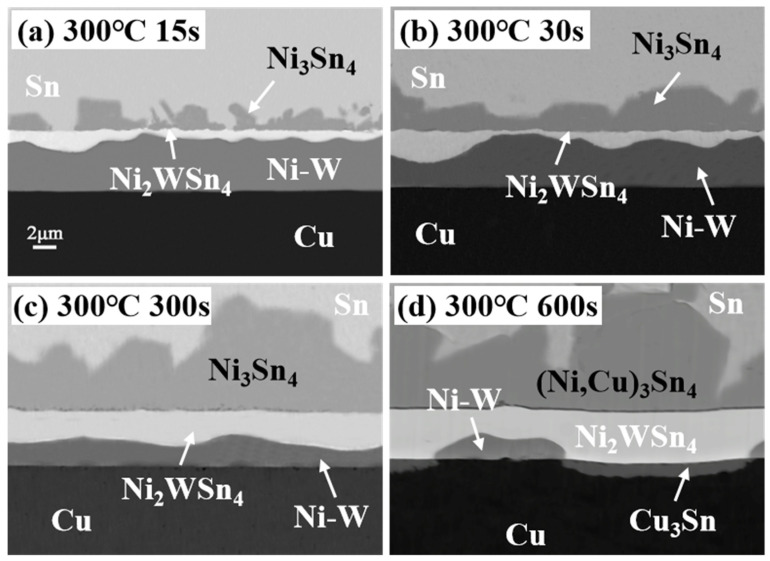
Cross-sectional SEM images of Sn/Ni-W/Cu substrates soldered for different periods of time at 300 °C: (**a**) 15 s; (**b**) 30 s; (**c**) 300 s; (**d**) 600 s.

**Figure 9:**
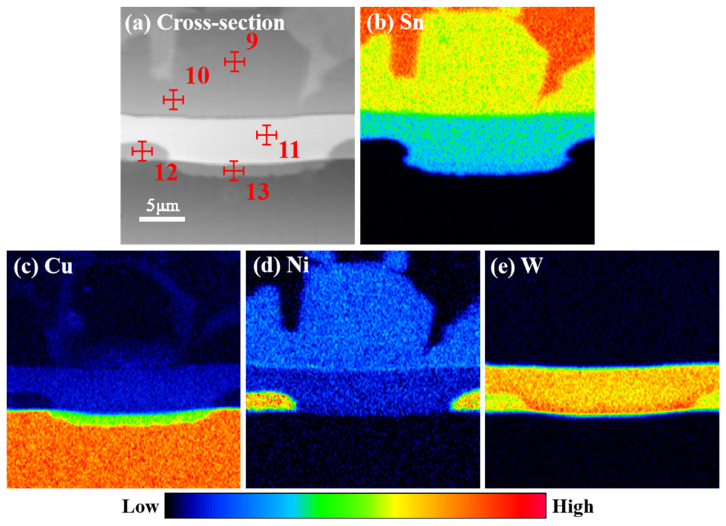
EDS maps of Sn/Ni-W/Cu substrates soldered for 600 s at 300 °C.

Figure 8 presents the cross-sectional SEM images of the Sn/Ni-W/Cu substrates subjected to soldering at 300 °C for varying periods. As illustrated in Figure 8, the cross-sectional microstructures of the Sn/Ni-W/Cu substrates soldered at 300 °C for 15, 30, and 300 s (Figure 8a–c) displayed markedly disparate characteristics in comparison to the cross-sectional microstructures of the Sn/Ni-W/Cu substrates soldered for 600 s (Figure 8d). Soldering at 300 °C for a period of less than 300 s resulted in the formation of an IMC comprising Ni_3_Sn_4_. Figure 9 depicts the EDS maps of the Sn/Ni-W/Cu substrates soldered for 600 s at 300 °C. The shift from dark blue to red in the graph reflects a gradual increase in concentration. In the Sn distribution diagram, the red color represents pure Sn, the yellow area represents (Ni,Cu)_3_Sn_4_, the green area represents the transition region between (Ni,Cu)_3_Sn_4_ and the “bright layer” Ni_2_WSn_4_, and the light blue represents the “bright layer” Ni_2_WSn_4_. In the Cu distribution diagram, this blue region depicts the reaction of (Ni,Cu)_3_Sn_4_ with Sn through the Ni-W diffusion barrier, the green color is Cu_3_Sn, and the orange color represents the Cu substrate. In the Ni distribution diagram, the light blue color represents (Ni,Cu)_3_Sn_4_, the dark blue color denotes the “bright layer” Ni_2_WSn_4_, and the orange color signifies the Ni-W layer. In the W distribution diagram, the orange area corresponds to the “bright layer” Ni_2_WSn_4_, while the yellow area represents the Ni-W layer. However, when the soldering time at 300 °C was 600 s, as illustrated in Figure 9 and Table 5, the (Ni,Cu)_3_Sn_4_ IMC was formed, in addition to a minor quantity of the Cu_3_Sn IMC. The varying Cu content at different locations resulted in the formation of a mixture of the (Cu_1_Ni_42_)_3_Sn_4_ and (Cu_3_Ni_40_)_3_Sn_4_ IMC. It can be observed that the Ni-W diffusion barrier layer lost its blocking function to inhibit the diffusion of Cu atoms when the interfacial reaction time was 600 s. The diffusion of Sn and Cu atoms through the IMC layer resulted in the formation of the (Ni,Cu)_3_Sn_4_ IMC and Cu_3_Sn IMC. The formation of voids as a result of the outward diffusion of Cu atoms has been demonstrated to have a deleterious effect on the reliability of solder joints [30,31].

### 3.3. Growth Kinetics Analysis of Interfacial Reactions

The interfacial growth kinetics of Sn/Cu solder joints can be expressed by Schaefer’s classical power rate empirical formula [32]. It can be demonstrated that the growth thickness of the interfacial IMC layer (the consumption thickness of the interfacial Ni-W diffusion barrier layer) and the soldering reaction time can be expressed by a power–exponential relationship equation.

(1) $$L=kt^{n}$$

The interfacial reaction time (*t*) of the soldering process is a function of the average thickness (*L*) of the Ni_3_Sn_4_-based IMC and Ni_2_WSn_4_ layers, the growth coefficient (*K*), and the growth index (*n*).

When *n* = 1, the growth conforms to the linear law and is therefore controlled by the interfacial reaction. When *n* = 1/2, the growth conforms to the parabolic law, and the growth is controlled by bulk diffusion. When *n* = 1/3, the growth conforms to the parabolic law, and the growth is controlled by grain boundary diffusion.

Upon taking logarithms on both sides of Equation (1), the following result is obtained:

(2) lnL=lnk+nlnt

**Figure 10:**
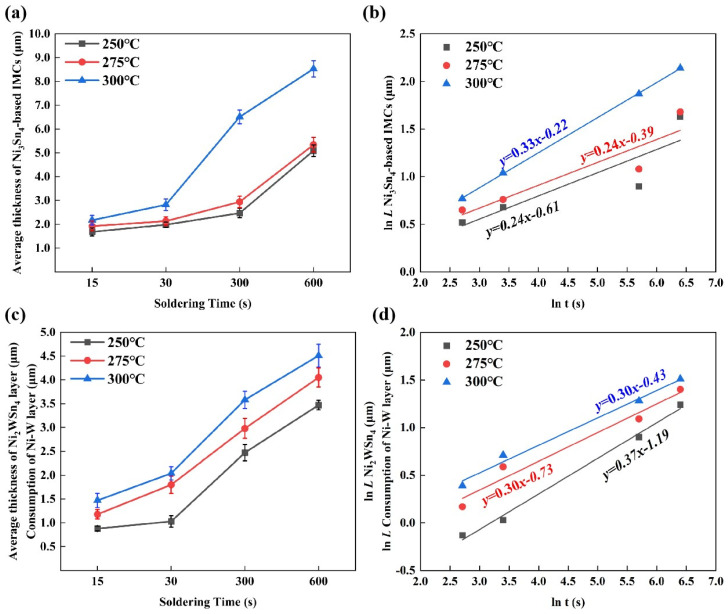
(**a**) The average thickness of Ni_3_Sn_4_-based IMCs. (**b**) The thickness of the Ni_3_Sn_4_-based IMCs as a function of soldering time at various temperatures on Sn/Ni-W/Cu substrates. (**c**) The average thickness of the Ni_2_WSn_4_ layer/consumption of the Ni-W diffusion barrier layer. (**d**) The average thickness of the Ni_2_WSn_4_ layer/consumption of the Ni-W diffusion barrier layer as a function of soldering time at various temperatures on Sn/Ni-W/Cu substrates.

Figure 10a presents the mean thickness of the Ni_3_Sn_4_-based IMCs obtained from Sn/Ni-W/Cu substrates subjected to distinct welding conditions. As illustrated in Figure 10a, the mean thickness of the interfacial Ni_3_Sn_4_-based IMCs of the Sn/Ni-W/Cu substrates acquired at 300 °C for periods of 300 and 600 s was markedly greater than that of the interfacial Ni_3_Sn_4_-based IMCs of Sn/Ni-W/Cu substrates obtained under alternative conditions. This may be attributed to the disparate diffusion mechanisms of Ni_3_Sn_4_-based IMC growth. Figure 10b presents a plot of the total thickness of the Ni_3_Sn_4_-based IMC layer versus the interfacial reaction time during the soldering process. At a soldering temperature of 250 °C, the growth coefficient n was 0.24, which indicates that the growth mode of the IMC was grain boundary diffusion. Nevertheless, the growth coefficients n were 0.24 and 0.33 for soldering temperatures of 275 °C and 300 °C, respectively, indicating that the growth of IMC is still controlled by grain boundary diffusion.

Figure 10c illustrates the thickness of the Ni_2_WSn_4_ layer (consumption of Ni-W diffusion barrier layer) in relation to different soldering conditions. As the temperature and duration of the soldering process increased, the average thickness of the Ni_2_WSn_4_ layer also increased. This is due to the fact that with the increase in soldering temperature and time, the dissolution of Ni and W elements in the liquid pure Sn solder increases, which is more favorable to the formation of Ni_2_WSn_4_. Figure 10d presents the average thickness of the Ni_2_WSn_4_ layer/consumption of the Ni-W diffusion barrier layer as a function of the Sn/Ni-W/Cu substrates’ soldering time at various temperatures. At a soldering temperature of 250 °C, the depletion coefficient n was 0.37, which is situated between 1/3 and 1/2. Consequently, the total thickness of the Ni_2_WSn_4_ layer (consumption of Ni-W diffusion barrier layer) was influenced by both the grain boundary diffusion and bulk diffusion. At a welding temperature of 275 °C, the growth coefficient (depletion coefficient) n was 0.30, while at a welding temperature of 300 °C, the growth coefficient (depletion coefficient) n was still 0.30, which was close to 1/3. Consequently, the total thickness of the Ni_2_WSn_4_ layer (consumption of Ni-W diffusion barrier layer) was controlled by grain boundary diffusion. As shown in Table 6, the growth diffusion rate of the Ni_3_Sn_4_-based IMC and the Ni_2_WSn_4_ layer (consumption diffusion rate of the Ni-W diffusion barrier layer) exhibited an increase with the rise in soldering temperature.

## 4. Conclusions

The objective of this study was to electrodeposit a Ni-W layer on a Cu substrate for the purpose of creating a diffusion barrier. Subsequently, the barrier properties between the Sn and Cu reactive couples were studied in a systematic and comprehensive manner. The main conclusions are as follows. The Ni-W layer has the capacity to impede the diffusion of Sn atoms into Cu. Furthermore, the Ni-W coating is a viable diffusion barrier at the Sn/Cu interface. The “bright layer” Ni_2_WSn_4_ could be observed in all Ni-W coatings during the soldering reflow process and its growth was almost linear. The structure of the Ni-W layer was such that it reduced the barrier properties that would otherwise be inherent to it. This was due to the “bright layer” Ni_2_WSn_4_ that covered the original Ni-W barrier layer. At a temperature of 300 °C for a duration of 600 s, the Ni-W barrier layer lost its blocking function. Once the “bright layer” Ni_2_WSn_4_ had completely covered the original Ni-W barrier layer, the diffusion activation energy for Sn diffusion into the Cu substrate side was significantly reduced, particularly in the area where the distortion energy was concentrated due to electroplating tension. Both the “bright layer” Ni_2_WSn_4_ and Sn grew rapidly, with the formation of Cu-Sn intermetallic compounds. At temperatures of 250 °C, the growth of Ni_3_Sn_4_-based IMCs was controlled by grain boundaries. Conversely, the growth of the Ni_2_WSn_4_ layer (consumption of Ni-W layer) was influenced by a combination of grain boundary diffusion and bulk diffusion. At temperatures of 275 °C and 300 °C, the growth of Ni_3_Sn_4_-based IMCs and the Ni_2_WSn_4_ layer (consumption of Ni-W layer) were both controlled by grain boundaries.

## Figures and Tables

**Table 1:** Chemical composition of the electrodeposition solution.

Material	NiCl_2_·6H_2_O	NiSO_4_·6H_2_O	Na_3_C_6_H_5_O_7_·H_2_O	Na_2_WO_4_·2H_2_O	C_6_H_4_SO_2_NNaCO·2H_2_O	C_12_H_25_SO_4_Na
Concentration	15 g/L	80 g/L	100 g/L	80 g/L	5 g/L	0.5 g/L

**Table 2:** Ni-W coating compositions corresponding to the indicated point in Figure 2.

Indicated Position	Element	wt.%	at.%
1	Ni	75.18	90.43
	W	24.82	9.57
2	Ni	74.61	90.29
	W	25.39	9.71

**Table 3:** Ni-W coating and IMC compositions corresponding to the indicated point in Figure 5.

Indicated Position	Element	wt.%	at.%
3	Cu	0.07	0.03
	Sn	74.36	57.11
	Ni	25.57	42.86
	W	0.00	0.00
4	Cu	0.00	0.00
	Sn	60.63	57.40
	Ni	16.41	28.59
	W	22.96	14.01
5	Cu	0.58	0.67
	Sn	1.55	0.96
	Ni	73.29	88.79
	W	24.58	9.58

**Table 4:** Ni-W coating and IMC compositions corresponding to the indicated point in Figure 7.

Indicated Position	Element	wt.%	at.%
6	Cu	0.08	0.04
	Sn	74.84	57.77
	Ni	23.78	42.19
	W	0.00	0.00
7	Cu	0.00	0.00
	Sn	60.91	57.86
	Ni	16.21	28.25
	W	22.88	13.89
8	Cu	0.57	0.93
	Sn	1.66	1.03
	Ni	72.87	88.18
	W	24.90	9.86

**Table 5:** Ni-W coating and IMC compositions corresponding to the indicated point in Figure 9.

Indicated Position	Element	wt.%	at.%
9	Cu	0.73	1.03
	Sn	75.13	57.41
	Ni	24.24	41.56
	W	0.00	0.00
10	Cu	1.82	2.56
	Sn	75.02	57.03
	Ni	23.16	40.41
	W	0.00	0.00
11	Cu	0.00	0.00
	Sn	60.28	57.46
	Ni	16.28	28.31
	W	23.44	14.23
12	Cu	0.53	0.87
	Sn	1.76	1.09
	Ni	72.68	88.13
	W	25.03	9.91
13	Cu	63.50	76.78
	Sn	36.46	23.2
	Ni	0.00	0.00
	W	0.04	0.02

**Table 6:** The diffusion rate (*k*) and kinetics time index (*n*) of the Sn/Ni-W/Cu substrates during the soldering process.

	Temperature (°C)	*k* (μm/s^1/2^)	*n*
Ni_3_Sn_4_-based IMCs (Growth)	250	0.54	0.24
	275	0.68	0.24
	300	0.80	0.33
Ni_2_WSn_4_ (Growth) Ni-W diffusion barrier layer (Consumption)	250	0.30	0.37
	275	0.48	0.30
	300	0.65	0.30

## Data Availability

The original contributions presented in the study are included in the article, further inquiries can be directed to the corresponding author.
